# Improving the efficiency of DNA extraction from iron incrustations and oilfield-produced water

**DOI:** 10.1038/s41598-024-53134-9

**Published:** 2024-02-05

**Authors:** Md Javed Foysal, Silvia J. Salgar-Chaparro

**Affiliations:** 1 Curtin Corrosion Centre, Western Australian School of Mines, Minerals and Energy, Curtin University, Bentley, WA Australia; 2 School of Environmental and Life Sciences, The University of Newcastle, Callaghan, NSW Australia; 3 Department of Genetic Engineering and Biotechnology, Shahjalal University of Science and Technology, Sylhet, Bangladesh

**Keywords:** Biological techniques, Computational biology and bioinformatics, Microbiology, Molecular biology

## Abstract

The quantity and quality of DNA isolated from environmental samples are crucial for getting robust high-throughput sequencing data commonly used for microbial community analysis. The differences in the nature and physicochemical properties of environmental samples impact DNA yields, and therefore, an optimisation of the protocols is always recommended. For instance, samples collected from corroded areas contain high concentrations of metals, salts, and hydrocarbons that can interfere with several steps of the DNA extraction protocols, thereby reducing yield and quality. In this study, we compared the efficiency of commercially available DNA extraction kits and laboratory-adopted methods for microbial community analysis of iron incrustations and oilfield-produced water samples. Modifications to the kits manufacturers’ protocols were included to maximise the yield and quality. For iron incrustations, the modified protocol for FastDNA Spin Kit for Soil yielded higher DNA and resulted in higher diversity, including the recovery of low-abundant and rare taxa in the samples, compared to DNeasy PowerSoil Pro Kit. The DNA extracted with modified phenol–chloroform methods yielded higher DNA but failed to pass quality control PCR for 16S sequencing with and without purification. The protocols mentioned here can be used to maximise DNA recovery from iron incrustations and oilfield-produced water samples.

## Introduction

Identification of microbes is crucial for finding solutions to many environmental problems. However, more than 99% of the bacteria in the environment are non-culturable and, therefore, cannot be detected through traditional culture-based methods^1,2^. Against this backdrop, the use of appropriate molecular methods with higher detection limits and superior sensitivity is a prerequisite for studying microbial communities in a particular environment^3^. Due to the incompetence of culture-based methods and the limit of detection, culture-independent high throughput sequencing (HTS) for the identification of microbial communities has recently gained much popularity^4,5^. HTS methods have helped to reveal the complexity of the spatial–temporal dynamics and diversity of microbial populations in different environments^6–8^. The process starts with the extraction of DNA directly from the environmental samples, followed by library preparation and HTS. The quality of DNA is crucial in generating high-quality data for HTS-based microbial community analysis.

DNA isolation from some environmental samples can be challenging due to the presence of humic acids, inhibitors, sediments, and contaminants that interfere with DNA extraction^9,10^. For instance, metal ions such as iron, zinc and tin can easily bind to template DNA through the formation of direct crosslink and inhibit PCR by blocking access of polymerase to the DNA^11–13^. In addition, the co-extraction of DNA from solid and liquid samples rich in chemicals and salts can reduce the quality of DNA for subsequent downstream applications^14^. Many studies reported optimisation of DNA extraction methods from environmental samples in the past three decades^15–19^; however, the type and diversity of environmental samples make it difficult to set a common method of DNA extraction. For instance, the basic principles for DNA extraction from solid and liquid samples are different and thus, the kits used for extractions. Furthermore, the addition of chemicals and modification of steps can result in high-concentration good-quality DNA from environmental samples^20^. Therefore, the choice of extraction methods and modifications have critical impacts on the DNA-based phylogenetics, diversity and abundance of any sample^21–23^. Considering the importance of DNA yield and quality for downstream applications such as PCR or HTS, selecting proper methods and optimising DNA extraction from metal-rich environments are equally important to reduce the burdens and biases from microbial community analysis.

In the energy sector, specifically in the hydrocarbon industry, microbes are associated with different types of environmental events. Among these, microbiologically influenced corrosion (MIC) is particularly complex to handle, as samples usually contain numerous inhibitors, including high concentrations of iron, salts, and hydrocarbons^24,25^. In MIC, microorganisms play an essential role in the deterioration of assets^26^; hence, assessing microbial diversity and understanding its functional capability is fundamental for preventing and mitigating undesired phenomena. Early MIC detection requires the identification of corrosive microorganisms in the system, which is currently conducted using DNA-based methods^3,27,28^. Currently, there are no standard protocols or established procedures for high-quality DNA extractions from corrosion-related samples; therefore, evaluating and optimising different extraction methods are paramount to obtaining meaningful data for MIC management. Successful extraction of nucleic acids will ensure the precise identification of microbial communities linked to corrosion, which will help to prevent MIC issues in the hydrocarbon industry.

This study aimed to improve the yield and quality of DNA from iron incrustations and produced water from Australian oilfields using commercial kits and manual extraction methods. We investigated whether the DNA extraction methods and modifications affect the diversity and composition of microbial communities identified through high-throughput sequencing of 16S rRNA gene.

## Results

### DNA yields

Both modified phenol–chloroform methods yielded a higher phenol-contaminated DNA but a lower 260/280 ratio. DNA recovery by method 1 (Barnett & Larson) was higher (322.4 ng/µl) but inferior (260/280 ratio: 1.18) than method 2 (Nishiguchi) (288.4 ng/µl, 260/280 ratio: 1.21). Both methods failed to pass the quality control in terms of 16S PCR and 1% agarose gel electrophoresis (Table S1).

#### In prepared samples compared to the community standard

For four iron incrustations, the DNA concentration obtained with each protocol is shown in Table S1 and DNA quality was checked in 1% agarose gel (Fig. S1). The highest DNA concentration was obtained with MP kit (Table S1, Fig. S1). Both phenol–chloroform methods were found inefficient in recovering good quality DNA from iron incrustations, and failed to amplify in PCR; therefore, no further analyses were conducted with these methods.

For produced water samples, heating incubation for 30 min yielded higher DNA compared to others without compromising the quality (Table S2). Consequently, 30 min of thermal treatment were included in all tested protocols. DNA concentrations obtained with each protocol are presented in Table S3. The PW kit yielded significantly lower DNA concentration compared to MP and PS kits. Similarly, the highest DNA concentration for water samples was obtained with the MP kit.

The extracted DNA was purified using PC kit, followed by amplification and sequencing of V3V4 regions of 16S rRNA gene. The comparative summarised results generated from sequence data for iron incrustation and water samples using qiime2 (SILVA 138) are shown in Table S4 and S5, respectively. It was observed that all extraction protocols were able to isolate DNA from eight microbial species present in the community standard; however, the overall results suggest that MP kit had better yield and quality as well as higher efficiency in extracting and recovering community DNA from both types of samples.

#### From field samples

DNA concentration obtained with each protocol is presented in Table S6 and S7. As observed in the previous stage, MP kit yielded a higher (*p*-value < 0.001) concentration of DNA (17.5 ± 1.4 ng/µl for iron incrustation 1, 586.4 ± 145.8 ng/µl for iron incrustation 2) than the PS kit (7.1 ± 1.2 ng/µl for iron incrustation 1, 35.9 ± 4.6 ng/µl for iron incrustation 2). Similar results were obtained for the oilfield water samples wherein MP (12.6 ± 2.1 ng/µl for oilfield water 1, 30.2 ± 1.4 ng/µl for oilfield water 2) yielded significantly (*p*-value < 0.001) higher concentration of DNA than PS kit (2.7 ± 1.8 ng/µl for oilfield water 1, 17.4 ± 2.6 ng/µl for oilfield water 2). The PCR amplification was found negative for all DNA samples extracted with MP and PS kits. A subsequent DNA clean-up was performed with PC kit and all samples showed positive amplification after purification (Fig. S2).

### Microbial diversity and composition in the field samples

#### Sequence statistics

Soil and water samples were represented by 1.1 million quality reads obtained from amplicon sequencing of 35 samples, ranging from 10,112 to 76,886 and an average of 28,436.5 ± 2678.2. After filtering, 1.1 million merged reads generated 1325 ASVs, 34 phyla, 162 orders, 258 genera. Each sample was sequenced at maximum depth to capture most of the diversity as revealed through a saturated rarefaction plot (Fig. S3) and high good coverage index values (Table S8).

#### Microbial diversity and composition in iron incrustations

**Figure 1:**
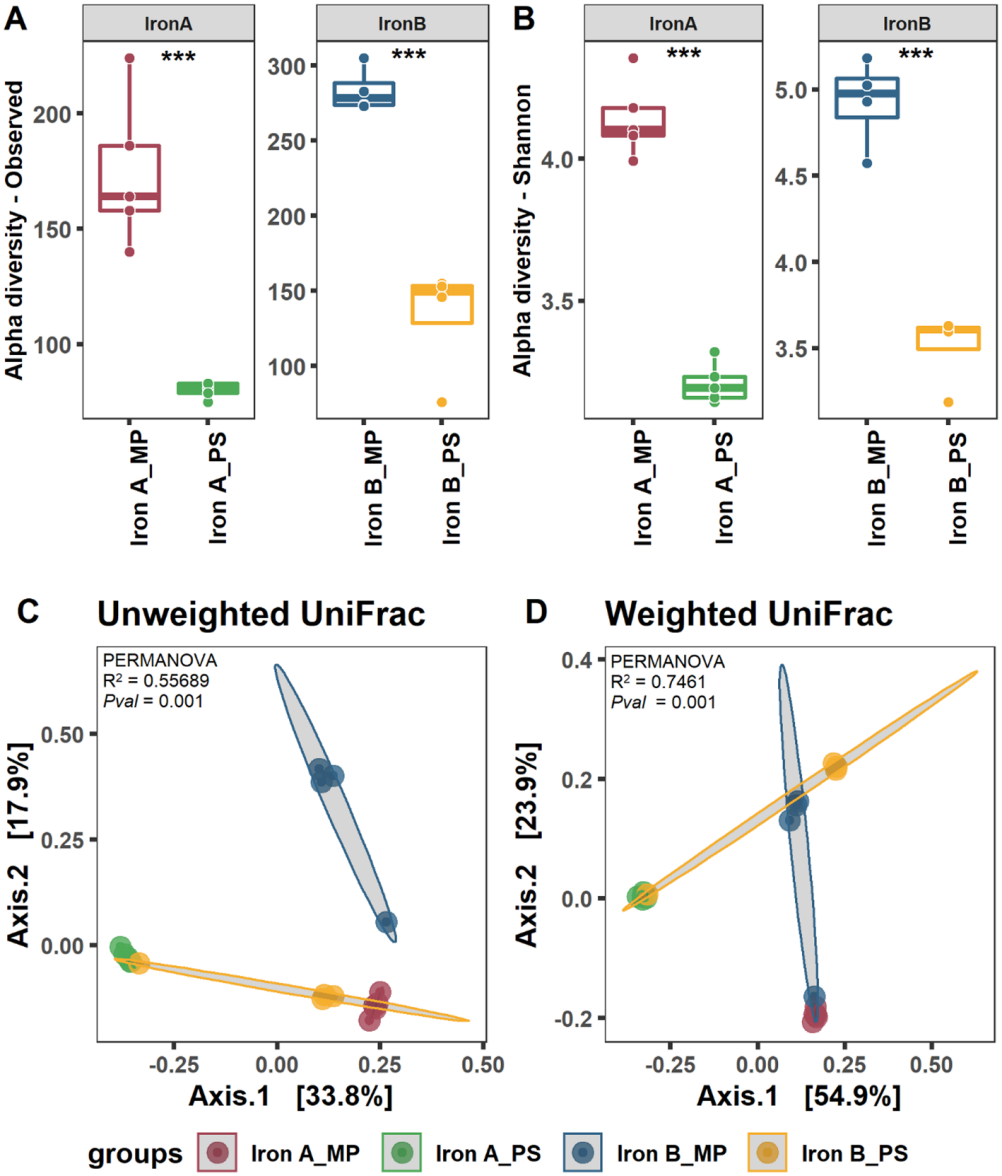
Diversity metrics of iron-incrustation samples. Alpha-diversity based on observed species (**A**) and Shannon index (**B**). Beta-diversity based on unweighted (**C**) and weighted (**D**) UniFrac distance metrics. The horizontal central lines in the box (**A**,**B**) showing the median values of the curated data. The box limits correspond to the 25th and 75th percentiles, and the whiskers are the 5th and 95th percentiles. ***Significant differences at α-level of 0.001.

Based on the observed species and Shannon as an indicator of alpha diversity measurements, MP kit captured significantly higher diversity in both iron A and B samples, compared to PS kit (Fig. 1A,B). Beta-ordination analysis showed that the selection of kit for iron incrustations’ DNA extraction had a significant influence on the identification of low-abundant rare and the richness of the most abundant bacterial communities. Principal coordinate analysis (PCoA) clearly separated microbial communities based on taxonomic dissimilarities (unweighted) and their relative abundance (weighted) as shown in Fig. 1C,D.

**Figure 2:**
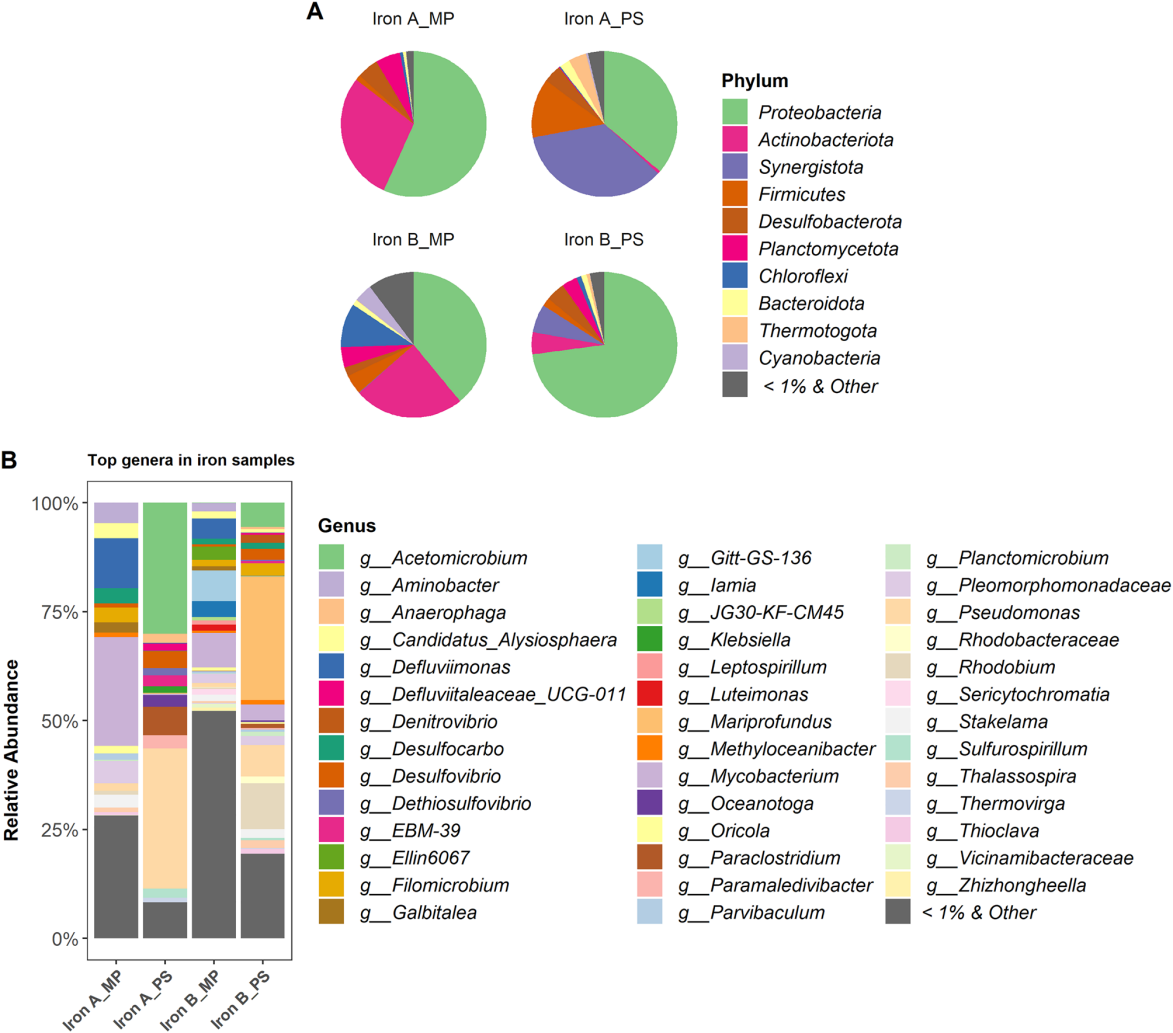
Relative abundance of bacteria in iron samples at phylum (**A**) and genus (**B**) level. Phyla and genera represent ≥ 1% of read abundance were considered for plotting. Less than 1% and unclassified were grouped as “other”.

Proteobacteria was found to be the most predominant phylum in iron incrustations extracted with both MP and PS kits. An inconsistency was observed for the second dominant phylum wherein MP favoured Actinobacteriota, and PS supported Synergistota. The second phylum Synergistota had a very high abundance with PS kit for iron B sample (Fig. 2A). At the genus level, we observed a similarity in microbial communities between samples A and B extracted with the same kit. Diverse bacterial communities were identified with MP kit despite having a significant percentage of bacteria with low abundance (< 1%) at the genus level. The iron sample A showed a higher abundance for *Mycobacterium* and *Defluviimonas* with MP, while *Pseudomonas* and *Acetomicrobium* were for PS kit (Fig. 2B).

#### Microbial diversity and composition in iron incrustations

**Figure 3:**
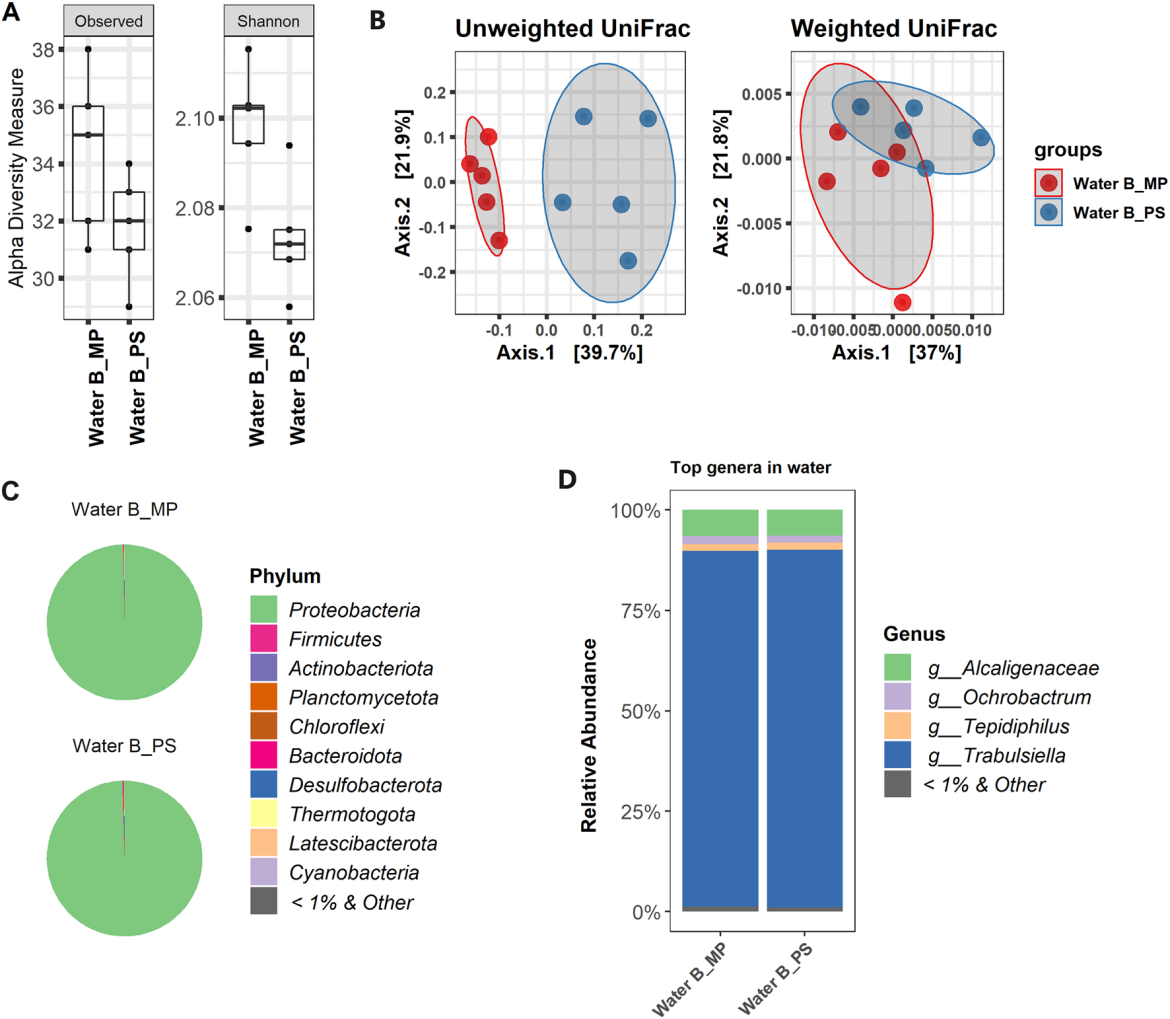
Diversity and composition of microbial communities in produced water samples from oilfield. (**A**) Alpha-diversity based on observed species and Shannon index. (**B**) Beta-diversity based on unweighted and weighted UniFrac distance metrics. The horizontal central lines in the box (for **A**) showing the median values of the curated data. The box limits correspond to the 25th and 75th percentiles, and the whiskers are the 5th and 95th percentiles. (**C**) Relative abundance at phylum level. (**D**) Relative abundance at genus level. Phyla and genera represent ≥ 1% of read abundance were considered for plotting. Less than 1% and unclassified were grouped as “other”.

Unlike soil samples, no difference between MP and PW was observed for water sample B in terms of bacterial alpha-diversity (Fig. 3A). Beta-ordinations, however, showed significant differences and the presence of new or rare species was found as the main driver linked to distinct separation with unweighted UniFrac distance metric (Fig. 3B).

For water, both kits generated similar community composition of bacteria dominated by Proteobacteria (> 95%) and *Trabulsiella* (> 80%) at phylum and genus levels, respectively (Fig. 3C,D).

## Discussion

DNA extraction from some environmental samples can be challenging but crucial in terms of performance, sustainability, and economy. Ample studies have been performed evaluating the efficacy of DNA extraction methods from water^29^, animal guts^30,31^, soil^21,32,33^, rock and coral^14,34^, freshwater and marine sediments^35,36^, and found variation in microbial communities. However, to our best knowledge, no study has yet been performed on evaluating and optimising DNA extraction from iron incrustations and produced water from oilfields. The proposed methods and modifications, therefore, could be used as a future reference for corroded samples from oilfields.

In this study, we used three of the most commonly used commercial kits for soil and water DNA extractions. We first evaluated the selectivity, specificity and accuracy of the commonly used commercial DNA extraction kits for soil and water in relation to a known microbial community (standard). Despite some variations in the relative abundance, no unwanted genera were classified from control iron powder samples that mixed with known microbial community standard. This result signifies that the used kits were accurate in the identification of microbial communities present in a sample, overruling the biases of spin filters reported earlier^37,38^. The accuracy of the tested kits in terms of percentages of DNA recovery for known genera from the community standard varied for the two kits. Interestingly, the DNeasy Power Water (PW) kit generated the most inferior results for water samples in terms of DNA yield and purity compared to the other two kits. Several factors could be responsible for these variations, including human gut-oriented bacteria in the microbial community standard and no bead-beating step in the power water kit. Bead beating is a critical step for the complete lysis of microbial cells during environmental DNA extraction, influencing diversity and composition more than non-bead beating methods^37,39,40^. This is more evident as MP kit recovered significantly higher Actinobacteriota, one of the largest Gram-positive bacterial phyla with the most diverse metabolic capabilities and ubiquitous in both terrestrial and aquatic environments^41–43^. Similar to phyla, at genus level, MP captured more ASVs for *Mycobacterium*, a Gram-positive environmental bacteria that can survive in extreme environments, specifically in metal-contaminated water^44^. Their role therefore can be crucial for the study of groundwater and soil contamination by heavy metals. Gram-positive bacteria have a thick cell wall that is difficult to lyse during DNA extraction compared to thin cell wall containing Gram-negative bacteria^45^. MP therefore generated a mixture of community containing both Gram-positive and Gram-negative bacteria. On the other hand, PS favoured Synergistota, Gram-negative bacteria predominantly found in anaerobic environments, including animal, gut soil, oil field, and wastewater treatment plants^46–48^. Consistent with some previous studies^23,37,49^, the efficacy of MP kit in yielding Gram-positive bacterial DNA from challenging environmental samples is found to be better than PS in this study. Considering the yield, quality and diversity of microbial communities, the bead-beating step is essential and recommended for all types of environmental DNA extractions.

The highest amount of DNA was recovered with MP kit outperforming PS and PW kits for soil and water, respectively. The higher yield of DNA in the present study with MP is possibly linked to unique glass beads in the lysing matrix, two different buffers, and binding matrix in comparison to other DNA extraction kits for environmental samples, as reported earlier^35,36,50^. Alongside DNA yield, the diversity and composition were also significantly higher with MP kit compared to PS for soil, suggesting more effective lysing of microbial cells during DNA extractions. Interestingly, more than 50% of reads for iron B samples and 25% of reads for iron A samples were unclassified with MP kit compared to significantly lower unclassified reads with PS kit, signifying identification of rare taxa by MP kit in the corroded samples. While this finding supports two other published reports from marine sediments and wastewater treatment plants^35,50^, it contradicts another study^36^ that reported no differences in observed OTUs and species in freshwater lake samples with different kits despite having higher DNA concentrations with MP kit. The type of samples might be associated with these differences as samples from wastewater treatment plants and marine sediments are quite complex and more challenging than freshwater lakes.

In the present study, phenol–chloroform extraction yielded higher concentration but poor quality DNA in terms of 260/280 ratio and showed negative results in 16S PCR and 1% agarose gel. This result upholds the complex nature of iron incrustation samples and produced water from oilfield, wherein quality is more important than quantity. Here, we collected samples from oilfields and corrosion sites rich in iron, manganese, and crude oil. Therefore, most of the samples were found negative in 16S PCR after extraction due to a poor 260/280 ratio and the presence of inhibitors. A higher 260/280 ratio, positive 16S PCR and greater recovery of microbial communities were ensured by an additional purification step with PowerClean following the extraction of samples. This further purification is reported to increase the yield of DNA and diversity of microbial communities from some challenging environmental samples such as oil-contaminated soil^51^, acidic soil^52,53^, soil from volcanic desert^54^, saline soil and water^55,56^, heavy metal contaminated soil^57^, and iron incrustations from mines^49,58^. Like previous reports, we found that the combination of DNA extraction and purification methods is very effective in removing PCR inhibitors from samples collected in challenging environmental conditions.

The overall data showed that the MP kit is better in characterising the low abundant and rare taxa in iron incrustations. PS kit on the other hand, was found very selective in capturing highly abundant or rich taxa in iron incrustations with low resolution for the unclassified communities. Nevertheless, the diversity in terms of richness was found to be significantly higher with the MP kit than the PS kit for iron incrustations. Even with water samples, the MP kit was ahead of PS kit in terms of unshared ASVs and species diversity. Both kits have a certain level of selectivity in DNA-based profiling of bacterial communities wherein the results can be influenced by the abundance of taxa as found in the weighted UniFrac distance metric. For instance, the MP kit showed lower sensitivity in detecting Gram-negative (-ve) bacteria for water samples. The overall findings also suggest that purification of DNA following extraction can increase DNA purity through the removal of inhibitors and, thereby, increasing the efficiency of 16S PCR and recovery of microbial communities in amplicon sequencing.

## Materials and methods

### Study design

**Figure 4:**
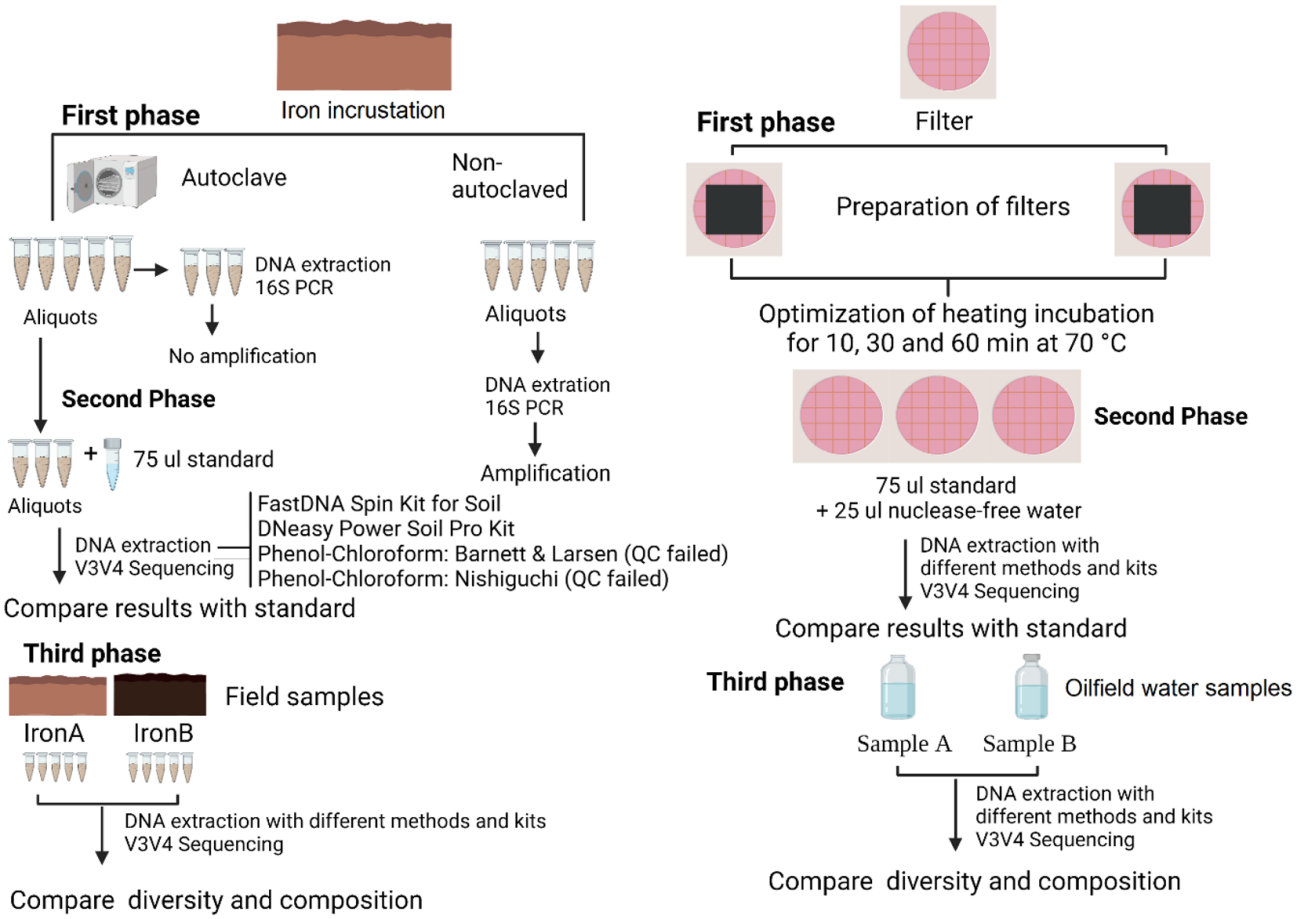
A flow diagram showing the study design and methods used for the data analysis.

A three-phase study was designed to evaluate the efficiency of commonly used DNA extraction methods and kits for soil and water samples from the perspective of iron incrustation samples and produced water from oilfield. An outline of the study design has been depicted in Fig. 4.

### Preparation of microbial standard

We first tested the influence of chemicals and metals on DNA yields and quality by preparing a mock sample with known microbial communities (ZymoBIOMICS) containing eight different species in various abundance. The aim was to test the accuracy of different methods in terms of microbial strain recovery from the standard from iron and water samples and to detect any kit-based contaminations (kit-ome) (Table S4 and S5).

### Preparation of iron samples

An iron incrustation sample received from an Australian oil production field was used to obtain the iron powder for the laboratory preparation of an iron incrustation sample. The iron powder was aliquoted in Eppendorf tubes (500 mg/tube) and autoclaved to degrade the DNA present in the sample. Following DNA extraction with FastDNA Spin kit for Soil (MP Biomedicals, USA), the DNA degradation by autoclaving was confirmed by the absence of DNA on 1% agarose gel and negative PCR amplification of bacterial 16S, 27F and 1492R^59^. The outcomes and efficiency of autoclaved samples were comparable to non-autoclaved ones that showed bands on the gel for DNA but not for 16S PCR (Table S9; Fig. S3).

### Amplification of iron samples

In the next step, 75 µl of microbial community standard (ZymoBIOMICS) was added to each of the four autoclaved iron powder tubes. The MP-extracted DNA samples were amplified with PCR using bacterial 16S primers covering the complete gene and V3-V4 hypervariable region. Only the positive control (DNA from *Shewanella* sp.) showed a band on the gel after the visualisation of PCR products (Fig. S5A). Considering positive DNA on gel but negative amplification in PCR, DNA purification from one sample per treatment (T2 and T3) was performed with the DNeasy PowerClean Clean-Up (PC) kit (Qiagen) to clean the sample from possible PCR inhibitors. The purified DNA sample (T3) from the non-autoclaved group showed a positive band on agarose gel, while the autoclaved sample (T2) was found negative (Fig. S5B).

### Preparation of water samples

Mock samples for screening of optimum incubation temperature were prepared by filtration of 200 ml of produced water from oilfield on each filter. The standard sample was produced by filtration of 25 ml of nuclease-free water mixed with 75 µl of ZymoBiomics Microbial Community standard.

### Optimisation of incubation for water samples

Heating is an optional step for DNA extraction from environmental samples. Thermal treatment of the samples during the isolation procedure helps to denature proteins and increase the speed of chemical reactions^60^. Nonetheless, heating may also lead to degraded DNA^61^. In this research, we evaluated three heating incubation times to select the optimal time that increases the yield of DNA without compromising its quality. DNA extraction from filter membranes was performed using 10, 30, and 60 min of heating incubation at 70 °C. DNA concentration and quality were used to select the optimal time.

### DNA extractions methods

DNA extraction was conducted using the following protocols: (i) Modified FastDNA Spin Kit for Soil: 3 × 3 cycles of homogenisation in FastPrep Ribolyser, extended centrifugation for 15 min at step 5, five minutes incubation at step 9, optional incubation (step 16) for 5 min at 55 °C. (ii) Modified DNeasy PowerSoil Pro Kit (Qiagen, Hilden, Germany): 3 × 3 cycles of homogenisation in FastPrep Ribolyser in place of vortexing at step 2. (iii) Modified DNeasy Power Water Kit (Qiagen, Hilden, Germany), only for water samples. (iv) Modified phenol–chloroform method reported by Barnett & Larson, 2012^62^: Resuspension of dried pellet with 100 µl TE buffer. (v) Modified phenol–chloroform method reported by Nishiguchi et al., 2002 with substantial modifications^63^ (Supplementary data M1.1). Following concentration measurements in Nanodrop spectrophotometer 2000 cc (Thermo Fisher Scientific, MA, USA), the DNA quality was checked in 1.5% agarose gel. The acronym MP, PS and PW were used to mention FastDNA Spin Kit for Soil, DNeasy PowerSoil Pro Kit, and DNeasy Power Water Kit, respectively. MP kit yielded the highest DNA for iron incrustations (Table S1, Fig. S1), and the modified PW kit generated poor data for prepared samples from the membrane filter compared to MP and PS (Table S3) and, therefore, not considered for field samples.

### Evaluation of DNA extraction methods using field samples

Two iron incrustation samples received from Australian oilfields were used in this phase to evaluate the extraction protocols with a more diverse community. These samples were collected from ballast tanks exhibiting internal corrosion defects. A total of 10 aliquots, each of 0.53 ± 0.007 mg were prepared from each sample. DNA was extracted in quintuplicate with the modified MP kit and the modified PS protocols described earlier. For water samples, two produced water samples received from Australian oilfields were used in this phase to evaluate the extraction protocols. Sample A was obtained from the high-pressure separator within an oil production facility, whereas, Sample B was collected from a wellhead of an oilfield. A total of eight membrane filters were prepared by filtering 200 ml of water. Each filter was equally divided, and DNA was extracted in quintuplicate with the modified MP and PS protocols. The concentration and quality of DNA were checked in agarose gel and qubit. For water, sample A extracted with PS failed to pass the required QC (16S PCR amplification) and was eventually removed for further analysis.

### PCR amplification, amplicon library preparation and sequencing

The amplicon for 16S V3V4 regions was generated in two-step PCR methods following “Illumina 16S metagenomic sequencing library preparation”^64,65^. The first PCR was run for 30 cycles, followed by beads purification of positive amplicons, and indexing via second PCR (12 cycles). Paired-end sequencing (v3 kit, 600 cycles) was performed with Illumina MiSeq platforms (Illumina Inc., CA, USA). The PCR conditions are available in supplementary data (Supplementary data M1.2).

### Data processing and statistical analysis

The raw sequence data was imported into qiime2 (v2021.4) for paired-end processing^66^. Quality trimming (denoising) of demultiplexed sequences was performed in q2-dada2 plug-in with the following parameters: -p-trim-left-l 0; -p-trunc-len-f 280; -p-trim-left-r 0; -p-trunc-len-r 220^67^. The DADA2 output as a feature frequency ASV (Amplicon Sequence Variants) table that represents biological features of amplicon sequence was classified taxonomically against SILVA 138 release using consensus blast^68^. The chimeric features and singletons were removed and the feature ASV table was filtered based on the lowest non-zero frequency 10. We removed chloroplast and mitochondrial sequences from the final data. Each sample was set to an even depth of 10,112 for downstream analysis of alpha–beta diversity and microbial composition. The rarefied ASV table, taxonomy table and metadata were used for alpha–beta diversity analysis with the R statistical software (v4.22)^69^. Observed species and Chao1 were chosen for the changes in diversity, while Shannon and Simpson's indices were used for the calculation of evenness among groups. Weighted (relative abundance) and unweighted (presence-absence) UniFrac distance metrics were used for the calculation of beta diversity. Alpha–beta diversity measurements were performed using phyloseq^70^, microbiomeSeq^71^, microbiome^72^ and vegan^73^ R packages in support of plotting packages ggplot2 . The relative abundance of bacteria at various taxa levels was calculated with the phyloseq R package. *Clostridium* sensu stricto 1 has been renamed as *Clostridium*. Kruskal–Wallis ranks test was used to compare alpha-diversity among groups. Centroid analysis of beta dispersion among the groups was performed as permutational multivariate analysis (PERMANOVA) with the vegan R package. Significantly abundant bacterial genera were identified using Linear Discriminant Analysis Effect Sizes (LefSe) at LDA cut-off value of 2.0 and more^74^. At every stage of data analysis, *p-value* of less than 0.05 was considered as statistically significant.

### Supplementary Information

Supplementary Information.

## Data Availability

The 16S rRNA sequences were deposited in the National Center for Biotechnology Information (NCBI) Sequence Read Archive under BioProject number PRJNA1004675.
