# Supplementary material for: Improving the efficiency of DNA extraction from iron incrustations and oilfield-produced water
Source: Sci Rep. 2024 Feb 5;14:2954. doi: 10.1038/s41598-024-53134-9 (PMC10844625; doi:10.1038/s41598-024-53134-9)
Supplement: Supplementary file 1 — Supplementary Information. [file 41598_2024_53134_MOESM1_ESM.docx]

**Supplementary data**

**Improving the efficiency of DNA extraction from iron incrustations and oilfield-produced water**

Md Javed Foysal^1,2,3^, Silvia Salgar Chaparro^1*^

^1^ Curtin Corrosion Centre, Western Australian School of Mines, Minerals and Energy, Curtin University, WA, Australia

^2^ School of Environmental and Life Sciences, The University of Newcastle, NSW, Australia

^3^ Department of Genetic Engineering and Biotechnology, Shahjalal University of Science and Technology, Sylhet, Bangladesh

ORCID

Md Javed Foysal – 0000-0002-2064-8897

Silvia J. Salgar-Chaparro – 0000-0002-7437-9725

*Corresponding author

[silvia.salgar@curtin.edu.au](about:blank)

**Table S1.** DNA concentration with the different extraction protocols for iron powder.

| Sample ID | Extraction protocol | DNA con. (ng/µl) | 260/280 ratio |
| --- | --- | --- | --- |
| T7 | FastDNA Spin Kit for Soil (MP Biomedicals) | 100 | 1.72 |
| T8 | DNeasy PowerSoil Pro Kit (Qiagen) | 13.2 | 1.58 |
| T9 | Phenol-chloroform method 1 (Barnett & Larson, 2012) | 322.4^⁋^ | 1.18 |
| T10 | Phenol-chloroform method 2 (Nishiguchi et al., 2002) | 288.4^⁋^ | 1.21 |

^⁋^ No band was detected on agarose gel for DNA.

**Table S2.** DNA concentration at different incubation temperatures for water samples

| Temperature (°C) | DNA concentration (ng/µl) | 260/280 ratio |
| --- | --- | --- |
| 10 | 12.8 | 1.68 |
| 30 | 28.9 | 1.81 |
| 60 | 24.6 | 1.41 |

**Table S3.** DNA concentration (ng/µl) with different extraction methods for water samples.

| Sample | DNeasy PowerWater Kit | FastDNA Spin Kit for Soil | DNeasy PowerSoil Pro Kit |
| --- | --- | --- | --- |
| Replicate 1 | 5.6 | 29.4 | 18.2 |
| Replicate 2 | 2.1 | 32.4 | 22.4 |
| Replicate 3 | 3.8 | 30.6 | 16.8 |

**Table S4.** Microbial profiling of prepared iron samples with different extraction methods.

| Genera | Relative abundance based on 16S rRNA sequencing | | |
| --- | --- | --- | --- |
|  | ZymoBIOMICS standard | FastDNA Spin Kit for Soil | DNeasy PowerSoil Pro Kit |
| *Lactobacillus* | 18.4 | 17.9 | 24.2 |
| *Bacillus* | 17.4 | 20.8 | 25.3 |
| *Staphylococcus* | 15.5 | 12.8 | 13.3 |
| *Listeria* | 14.1 | 11.8 | 12.7 |
| *Salmonella* | 10.4 | 13.1 | 8.8 |
| *Escherichia* | 10.1 | 15.1 | 9.4 |
| *Enterococcus* | 9.9 | 5.1 | 4.1 |
| *Pseudomonas* | 4.2 | 3.4 | 2.2 |

**Table S5.** Microbial profiling for membrane filter samples obtained with different extraction methods.

| Genera | Relative abundance based on 16S rRNA sequencing | | |  |
| --- | --- | --- | --- | --- |
|  | ZymoBIOMICS standard | FastDNA Spin Kit for Soil | DNeasy PowerSoil Pro Kit | DNeasy PowerWater Kit |
| *Lactobacillus* | 18.4 | 20.1 | 24.0 | 27.1 |
| *Bacillus* | 17.4 | 33.5 | 31.0 | 33.7 |
| *Staphylococcus* | 15.5 | 19.2 | 14.1 | 12.5 |
| *Listeria* | 14.1 | 15.7 | 12.1 | 17.2 |
| *Salmonella* | 10.4 | 3.2 | 5.6 | 1.1 |
| *Escherichia* | 10.1 | 2.6 | 6.3 | 1.9 |
| *Enterococcus* | 9.9 | 4.9 | 5.2 | 6.2 |
| *Pseudomonas* | 4.2 | 0.7 | 1.6 | 0.3 |

**Table S6.** DNA concentration with different extraction methods (field samples).

| Sample | DNA concentration (ng/µl) | | | |
| --- | --- | --- | --- | --- |
|  | Iron A | | Iron B | |
|  | FastDNA Spin Kit for Soil | DNeasy PowerSoil Pro Kit | FastDNA Spin Kit for Soil | DNeasy PowerSoil Pro Kit |
| Replicate 1 | 18.7 | 3.2 | 110.2 | 26.8 |
| Replicate 2 | 17.5 | 5.4 | 851.3 | 52.6 |
| Replicate 3 | 15.2 | 9.0 | 927.8 | 35.4 |
| Replicate 4 | 14.0 | 8.4 | 564.5 | 28.6 |
| Replicate 5 | 22.1 | 9.0 | 478.4 | 36.2 |

**Table S7.** DNA concentration with different extraction methods (field samples).

| Sample | DNA concentration (ng/µl) | | | |
| --- | --- | --- | --- | --- |
|  | Water 1 | | Water 2 | |
|  | FastDNA Spin Kit for Soil | DNeasy PowerSoil Pro Kit | FastDNA Spin Kit for Soil | DNeasy PowerSoil Pro Kit |
| Replicate 1 | 11.9 | 2.4 | 29.4 | 17.8 |
| Replicate 2 | 10.7 | 1.5 | 30.3 | 23.6 |
| Replicate 3 | 14.3 | 1.8 | 30.6 | 15.3 |
| Replicate 4 | 13.6 | 5.1 | 30.2 | 12.8 |

**Table S8.** Good’s coverage index value for 35 samples

| SampleID | Groups | Source | Good's coverage |
| --- | --- | --- | --- |
| T016 | Iron A_PS | Iron incrustation | 0.998 |
| T017 | Iron A_PS | Iron incrustation | 0.997 |
| T018 | Iron A_PS | Iron incrustation | 0.998 |
| T019 | Iron A_PS | Iron incrustation | 0.998 |
| T020 | Iron A_PS | Iron incrustation | 0.996 |
| T021 | Iron A_MP | Iron incrustation | 0.996 |
| T022 | Iron A_MP | Iron incrustation | 0.998 |
| T023 | Iron A_MP | Iron incrustation | 0.998 |
| T024 | Iron A_MP | Iron incrustation | 0.996 |
| T025 | Iron A_MP | Iron incrustation | 0.998 |
| T026 | Iron B_PS | Iron incrustation | 0.998 |
| T027 | Iron B_PS | Iron incrustation | 0.997 |
| T028 | Iron B_PS | Iron incrustation | 0.998 |
| T029 | Iron B_PS | Iron incrustation | 0.998 |
| T030 | Iron B_PS | Iron incrustation | 0.998 |
| T031 | Iron B_MP | Iron incrustation | 0.998 |
| T032 | Iron B_MP | Iron incrustation | 0.998 |
| T033 | Iron B_MP | Iron incrustation | 0.997 |
| T034 | Iron B_MP | Iron incrustation | 0.998 |
| T035 | Iron B_MP | Iron incrustation | 0.998 |
| T001 | Water A_MP | Water | 0.997 |
| T002 | Water A_MP | Water | 0.995 |
| T003 | Water A_MP | Water | 0.996 |
| T004 | Water A_MP | Water | 0.997 |
| T005 | Water A_MP | Water | 0.995 |
| T006 | Water B_MP | Water | 0.992 |
| T007 | Water B_MP | Water | 0.995 |
| T008 | Water B_MP | Water | 0.996 |
| T009 | Water B_MP | Water | 0.995 |
| T010 | Water B_MP | Water | 0.994 |
| T011 | Water B_PS | Water | 0.998 |
| T012 | Water B_PS | Water | 0.996 |
| T013 | Water B_PS | Water | 0.997 |
| T014 | Water B_PS | Water | 0.997 |
| T015 | Water B_PS | Water | 0.998 |

**Table S9.** The DNA concentration in iron powder

| Sample ID | DNA concentration (ng/µl) |
| --- | --- |
| T1 (autoclaved tube) | Not detected |
| T2 (autoclaved tube) | Not detected |
| T3 (non- autoclaved tube) | 12.4 |
| T4 (non-autoclaved tube) | 9.3 |


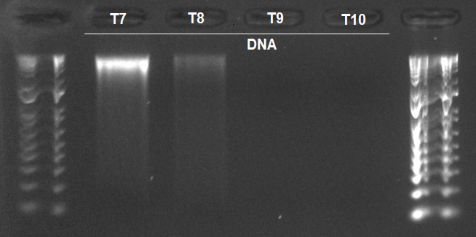


**Fig. S1.** Visualization of genomic DNA on 1.5% agarose gel


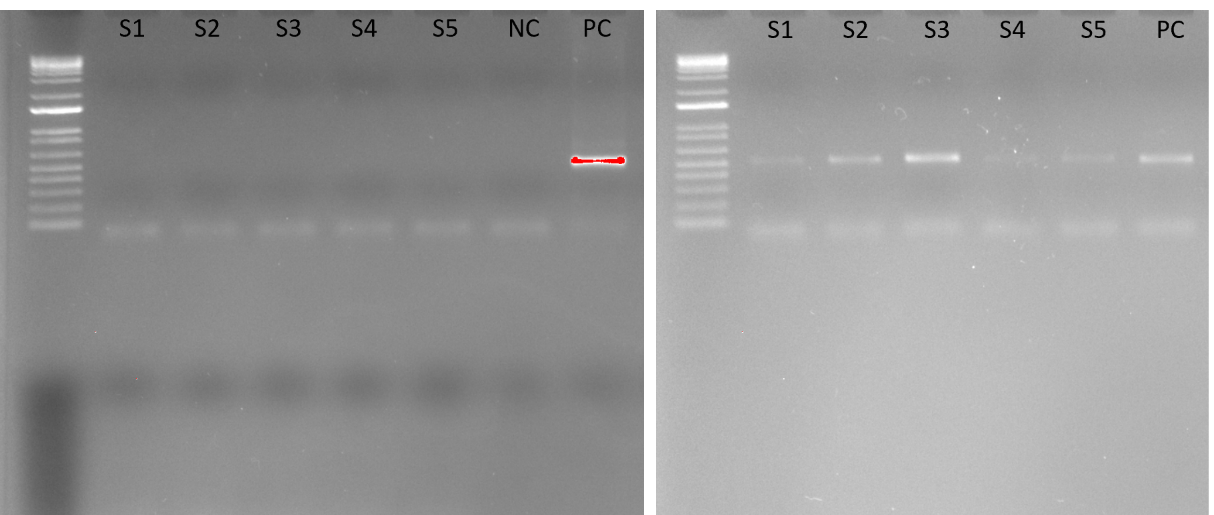


**Fig. S2**. PCR amplification of V3V4 for the impure (left) and pure (right) DNA from iron-rich samples extracted with Power Soil Pro Kit.


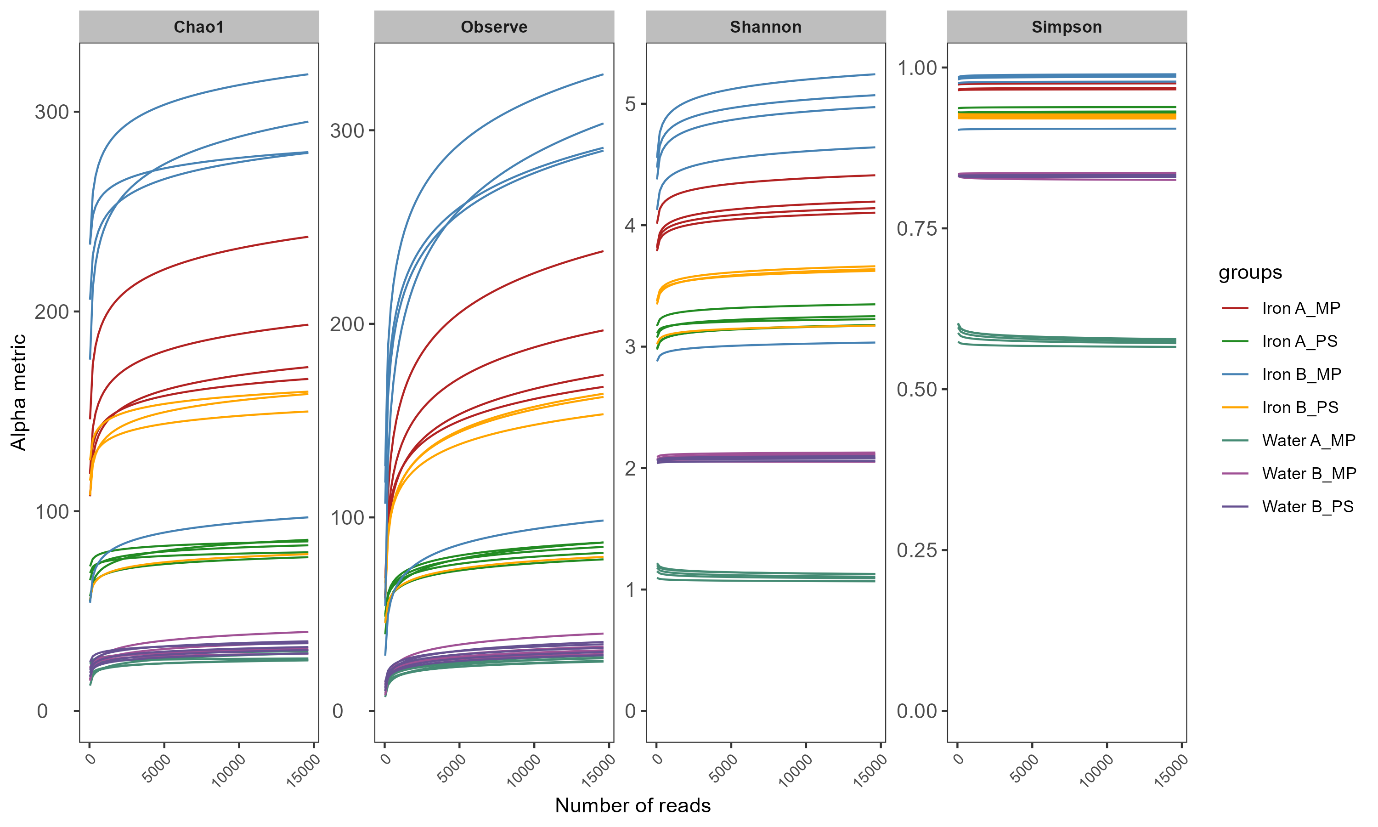


**Fig. S3**. Rarefaction curve showing the depth and saturation level of sequences for 35 samples from seven different groups.


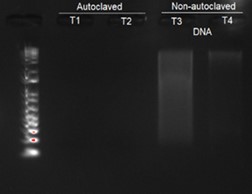


**Fig. S4.** Visualization of genomic DNA on 1.0% agarose gel


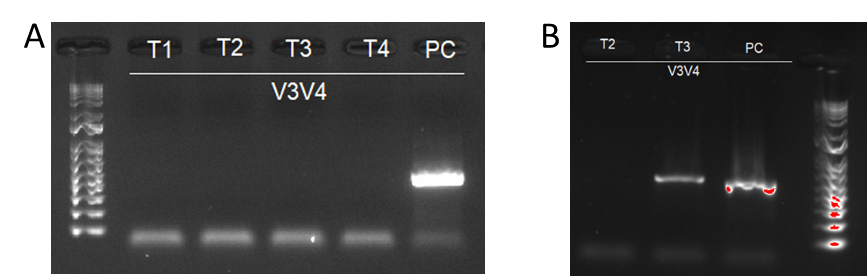


**Fig. S5.** Visualization of 16S rRNA amplicons on 1.0% agarose gel. Non-purified DNA (A); purified DNA (B). PC: positive control.

**Supplementary data: Methods (M1)**

***M1.1. Modified phenol-chloroform method***

In brief, 250 mg of soil sample was mixed individually with 1 mL of InhibitEX buffer and incubated at 95°C for 5 min. After centrifugation at 14,000 x g for 5 min, the resultant supernatant was transferred to a new tube, followed by the addition of 600 µL buffer AL and 25 µL of proteinase K and incubation for 1 h at 70 °C. The cell lysate was thoroughly mixed with one volume of phenol:chloroform: isoamyl alcohol solution (25:24:1) for 1 min and centrifuged at 10,000 x g for 5 min. After recovering the aqueous phase, the process was repeated. Subsequently, the aqueous phase was transferred into a new 1.5 mL tube before adding an equal volume of chloroform: isoamyl alcohol solution (24:1). The mixture was vortexed for 1 min and centrifuged at 10,000 x g for 5 min. The aqueous phase was again transferred into a new 1.5 mL tube, after which was added with two volumes of ice-cold 95% (v/v) ethanol to precipitate the DNA. The resulting DNA pellet was washed with 70% (v/v) ethanol and resuspended in 50 µL Tris-EDTA buffer (10 mM Tris-HCL, pH 8.0, 1 mM EDTA).

***M1.2. PCR conditions***

PCR was carried out in a final volume of 50 µl containing 25 µl Hot Start 2X Mater Mix (New England BioLab Inc., USA), 2 µl of template DNA, 1 µl of each V3 and V4 primers for bacteria, and 21 µl of nuclease-free water. PCR conditions were used as follows: an initial denaturation of 5 min at 95 °C, followed by 34 cycles of 30 s at 95 °C, 55 C for 40 s, 30 s at 72 °C, 72 °C for 30 s, and final extension of 72 °C for 10 min. The second PCR was performed with the same conditions but for only 12 cycles.
